# *Moniliophthora perniciosa* Necrosis- and Ethylene-Inducing Protein 2 (MpNep2) as a Metastable Dimer in Solution: Structural and Functional Implications

**DOI:** 10.1371/journal.pone.0045620

**Published:** 2012-09-24

**Authors:** Guilherme A. P. de Oliveira, Elen G. Pereira, Cristiano V. Dias, Theo L. F. Souza, Giulia D. S. Ferretti, Yraima Cordeiro, Luciana R. Camillo, Júlio Cascardo, Fabio C. Almeida, Ana Paula Valente, Jerson L. Silva

**Affiliations:** 1 Programa de Biologia Estrutural, Instituto de Bioquímica Médica, Instituto Nacional de Biologia Estrutural e Bioimagem, Centro Nacional de Ressonância Magnética Nuclear Jiri Jonas, Universidade Federal do Rio de Janeiro, Rio de Janeiro, Brazil; 2 Laboratório de Proteômica, Centro de Biotecnologia e Genética, Universidade Estadual de Santa Cruz, Ilhéus, Bahia, Brazil; 3 Mars Center For Cocoa Science, Itajuípe, Bahia, Brazil; 4 Faculdade de Farmácia, Universidade Federal do Rio de Janeiro, Rio de Janeiro, Brazil; University of South Florida College of Medicine, United States of America

## Abstract

Understanding how Nep-like proteins (NLPs) behave during the cell cycle and disease progression of plant pathogenic oomycetes, fungi and bacteria is crucial in light of compelling evidence that these proteins play a role in Witches` Broom Disease (WBD) of *Theobroma cacao*, one of the most important phytopathological problems to afflict the Southern Hemisphere. The crystal structure of MpNep2, a member of the NLP family and the causal agent of WBD, revealed the key elements for its activity. This protein has the ability to refold after heating and was believed to act as a monomer in solution, in contrast to the related homologs MpNep1 and NPP from the oomyceteous fungus *Phytophthora parasitica*. Here, we identify and characterize a metastable MpNep2 dimer upon over-expression in *Escherichia coli* using different biochemical and structural approaches. We found using ultra-fast liquid chromatography that the MpNep2 dimer can be dissociated by heating but not by dilution, oxidation or high ionic strength. Small-angle X-ray scattering revealed a possible tail-to-tail interaction between monomers, and nuclear magnetic resonance measurements identified perturbed residues involved in the putative interface of interaction. We also explored the ability of the MpNep2 monomer to refold after heating or chemical denaturation. We observed that MpNep2 has a low stability and cooperative fold that could be an explanation for its structure and activity recovery after stress. These results can provide new insights into the mechanism for MpNep2′s action in dicot plants during the progression of WBD and may open new avenues for the involvement of NLP- oligomeric species in phytopathological disorders.

## Introduction

*Moniliophthora perniciosa* is a basidiomycete with a hemibiotrophic life cycle [1], [2] that causes Witches` Broom Disease (WBD) in *Theobroma cacao*. This pest has brought about agro-economic losses in all of the cacao-growing regions in South America. The disease begins with the ability of basidiospores to infect meristematic cacao tissues including shoots, flowers and young developing fruits. Infection occurs through airborne dispersal in high-humidity environments [3], usually at night. Initially, the density of the fungal mycelium in the plant is very low [4], characterizing the biotrophic growth phase. After 1–2 months, tissue death occurs, forming a dry broom [5]. The proliferation of the fungus and the colonization of attached necrotic tissues lead to morphological changes in the hyphae, characterizing the saprotrophic phase. Subsequent spore formation can occur on any infected necrotic tissue, thus completing the disease cycle.

*Moniliophthora perniciosa* Nep2 (MpNep2) belongs to a family of microbial proteins that are secreted by various pathogens, including plant pathogenic oomycetes, fungi and bacteria [6]. Initially identified from the culture filtrate of *Fusarium oxysporum* [7], necrosis- and ethylene-inducing peptide 1 (NEP1)-like proteins (NLPs) are small proteins of ∼24 kDa that exhibit a high degree of sequence conservation. A conserved heptapeptide (GHRHDWE) and some conserved cysteine residues are present in all NLP sequences and are involved in their activities and the formation of disulfide bridges, respectively [8]–[11]. NLPs are classified as type I or type II depending on whether there are two or four cysteine residues at conserved positions [9]. NLPs trigger defense-associated responses when the protein is targeted to the apoplastic side of dicot cells, and cell death ensues in numerous dicot species but not in monocots [7], [8], [12], [13], [14]. Necrotic activity can be triggered by the release of ethylene [12], [15], [16], increased production of superoxide anions and accumulation of defense-related transcripts. Studies of the oomycete *Pythium aphanidermatum* showed that these proteins can also induce programmed cell death (PCD) [8], [13], [14], [17], [18]. However, the exact mechanism by which NLPs lead to plant death is unclear. Necrosis induction is not always accompanied by ethylene production [7], [15], indicating that other mechanisms may be involved.

NLP_Pya_ from *Pythium aphanidermatum* was previously shown to be a toxin that exerts its function by mediating membrane disruption in dicot plants [11]. A model of NLP interaction with the host`s membrane was proposed based on structural similarities between NLPs and pore-forming toxins known as actinoporins isolated from marine invertebrates [19]. The membrane disruption activity of NLPs alerts the plant's immune system, leading to the release of host-derived endogenous elicitors and consequently plant cell death. No sign of interaction between MpNep2 and lipids was shown by electron paramagnetic resonance or fluorescence measurements, suggesting that MpNep2 requires other anchoring elements from the membrane to promote cytolysis [20]. Additionally, cell death was reported to occur in a Ca^2+^-rich environment for NLP_Pya_. Scavenging extracellular calcium using a membrane-impermeable Ca^2+^-chelator abolishes the plasma membrane-disintegrating activity of NLPs, suggesting that the binding of a Ca^2+^ ion is crucial for NLP function [11]. However, in contrast to the results with NLP_Pya_, MpNep2 does not depend on an ion to accomplish its necrosis activity [20].

The presence of multiple NLPs in various phytopathogenic microorganisms suggests a significant role of these proteins in pathogenicity. The over-expression of Nep1 in the hypovirulent fungus *Colletotrichum coccodes* dramatically increases its aggressiveness toward the host plant and even enlarges the host range of this pathogen [21]. This evidence strongly suggests that NLPs may act as a virulence factor by facilitating the colonization of host tissues during the saprotrophic phase of pathogen growth [22]. Five NLP members were identified in the genome of *M. perniciosa* (MpNep), although a recent report showed that MpNep2 is the only NLP expressed during the fungal infection [20]. Moreover, differences in the physical properties of MpNep isoforms show that, in contrast to the heat-labile characteristic of most NLPs, MpNep2 refolds after heating and recovers its ability to induce necrosis in plant cells [23]. Its unusual ability to recover necrotic activity in tobacco leaves was shown even after exposure to 100°C for 30 min. MpNep1, with a highly similar sequence, and NPP protein, an NLP from *Phytophthora parasitica*, were also assessed in this experiment; in contrast to MpNep2, both proteins precipitated at approximately 40°C and were unable to cause necrosis after heating [23]. Finally, in solution under non-denaturing and denaturing conditions, MpNep1 and NPP appear to exist as oligomers, whereas MpNep2 is believed to exist only as a monomer [23]. Because MpNep2, the causal agent of WBD in *Theobroma cacao*, has different physical properties compared to related proteins and because WBD has a severe agro-economic impact, the primary goal of this work is to characterize MpNep2′s oligomerization state and its ability to refold after heating. The biochemical and structural characterization of an NLP dimer is an important challenge for understanding its functionality in WBD and other phytopathological diseases in light of sequence similarities within the NLP family.

## Materials and Methods

### Protein preparation

The MpNep2 sequence was cloned into the pET28a vector (Novagen, San Diego, CA) between *BamHI/HindIII* sites (Promega Corporation) to yield an HIS-tagged protein. After purification, a cloning artifact of 15 residues (GSHMASMTGGQQMGR) remained in the N-terminus region of the MpNep2 sequence. The plasmid was transformed into *Escherichia coli* BL21(DE3) cells, which were then plated on LB agar with kanamycin (50 µg/mL) and grown overnight at 37°C. Cultures were grown to an A_600nm_ of 0.8 at 37°C and cooled with shaking at 22°C prior to induction for 16 h at 22°C with 1 mM isopropyl-1-thio-β-D-galactopyranoside (IPTG). Cells were harvested by 15 min of centrifugation at 10,000 x*g* at 4°C and stored at −80°C or resuspended in 25 mL of 20 mM phosphate buffer (pH 7.4) containing 500 mM NaCl, 1% Tween 20 and 1 mM of the protease inhibitor PMSF (lysis buffer). Cells were sonicated, and insoluble proteins and cell debris were harvested through a 20 min centrifugation at 10,000 x*g* at 4°C. The supernatant was filtered through a 0.22 µm PVDF membrane (Whatman, GE Healthcare) and loaded onto a HisTrap FF affinity column (GE Healthcare) equilibrated with 20 mM phosphate buffer (pH 7.4) containing 500 mM NaCl (buffer A). The loaded column was washed with buffer A until baseline stabilization was reached. Weakly bound proteins were eluted with 5 column volumes of 5% buffer B (buffer A plus 0.5 M imidazole) followed by MpNep2 elution with a linear gradient of 5–50% buffer B. Peak fractions were analyzed by SDS-PAGE, concentrated, and loaded onto a Sephacryl 16/60 S-100 size-exclusion column (GE Healthcare) equilibrated with 20 mM phosphate buffer (pH 7.4) containing 80 mM NaCl. Thrombin cleavage (1–5 μg for 5–8 h at 4°C) was performed at this step to produce a non-tagged MpNep2. Analytical size exclusion on a Superdex 75 column (GE Healthcare) confirmed that MpNep2 was eluted at the expected volume for monomeric and dimeric protein. Fractions containing pure protein were pooled and concentrated in Vivaspin 20, 10,000 MWCO (GE Healthcare). The concentration of the protein was determined by absorbance spectroscopy at 280 nm using a calculated extinction coefficient of 53,065 M^−1^.cm^−1^ for the monomer and 106,130 M^−1^.cm^−1^ for the dimer. Measurements were performed in 20 mM phosphate buffer (pH 7.4) containing 80 mM NaCl at 25°C using a UVMini-1240 Shimadzu system. All columns were used in an Äkta system (GE Healthcare). For resonance assignment, proteins were isotopically ^15^N and ^15^N^13^C-D_2_O labeled; ^15^N labeling was used for chemical shift perturbation analysis only. Labeled proteins were purified as described above.

### Ultra-fast liquid chromatography (UFLC)

UFLC was performed in a Shimadzu system using a prepackaged Superdex 75 or Superdex 200 column (Pharmacia). The standard running buffer was 20 mM phosphate buffer (pH 7.4) containing 80 mM NaCl. The corresponding urea concentrations used in UFLC experiments are depicted in the figure legends. A flow rate of 0.7 mL.min^−1^ was used. Samples were monitored by measuring their absorbance at 280 nm and Trp fluorescence emission (not shown) at 340 nm (excitation at 280 nm).

### Mass spectrometry (MS/MS)

The MpNep2 monomer and dimer were subjected to tryptic digestion (0.01 μg.μL^−1^) in 25 mM NH_4_HCO_3_ at 37°C, and peptide mixtures were analyzed by online nanoflow liquid chromatography tandem mass spectrometry (LC-MS/MS) on a nanoAcquity system (Waters, Milford, MA) coupled to a Q-Tof micro Mass Spectrometer (Waters). Peptide mixtures were loaded onto a 1.7-μm ×100-mm nanoAcquity UPLC BEH300 column packed with C18 resin (Waters) and separated at a flow rate of 0.6 μL.min^−1^ using a linear gradient of 1 to 50% solvent B (95% acetonitrile with 0.1% formic acid) for 23 min, followed by a sharp increase to 85% B over 4 min, and held at 85% B for another 3 min. Solvent A was 0.1% formic acid in water. The effluent from the UPLC column was electrosprayed directly into the mass spectrometer. The instrument was operated in data-dependent mode to automatically switch between full scan MS and MS/MS acquisition. All MS and MS/MS raw data were processed in Masslynx v4.1 (Waters) and searched against a target protein sequence database (Swissprot or NCBInr) using the Mascot server v4.1 (http://www.matrixscience.com/). Search criteria were as follows: trypsin digestion; variable modifications set as carbamidomethyl (Cys) and oxidation (Met); max of one missed cleavage allowed; and a peptide mass tolerance of ±0.3 Da for the parent ion and 0.10 Da for the fragment ions.

### Cross-linking experiments

Purified MpNep2 monomer and dimer in 20 mM phosphate buffer (pH 7.4) containing 80 mM NaCl were subjected to chemical cross-linking with a glutaraldehyde concentration of 0.1%. Cross-linking reactions were performed with equal amounts of MpNep2 monomer and dimer (10, 40 and 100 μM for the monomer and 5, 20 and 50 μM for the dimer) at 37°C for 5 min and stopped with 1 M Tris-Cl (pH 8.0). Cross-linked proteins were visualized using 15% SDS-PAGE stained with G-250 Coomassie brilliant blue.

### Fluorescence spectroscopy

Fluorescence emission measurements were recorded on an ISSK2 spectrofluorometer (ISS Inc., Champaign, IL). Trp residues were excited at 280 nm, and emission was recorded from 300 to 400 nm. Changes in the fluorescence spectra in the presence of each concentration of urea (u) were quantified as the center of spectral mass (*v*_u_) in cm^−1^ (equation 1):
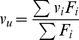
(1)where *F*_i_ represents the fluorescence emitted at wavenumber *v*_i_ and the summation is carried out over the range of appreciable values of *F*. The degree of denaturation (*α*) in a given condition was calculated from the changes in the center of spectral mass (*v*) in that condition (equation 2):
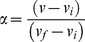
(2)where vi is the initial value of the center of spectral mass (folded protein) and vf is the final value (unfolded protein). For thermodynamic parameters, the free energy change can be obtained empirically using equation 3 [24], [25]:
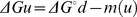
(3)where ΔGu is the free energy of denaturation at each urea concentration (u), ΔG°d is the free energy of denaturation in the absence of denaturant, and (m) is a constant proportional to the difference in solvent-accessible surface area as the protein unfolds. The free energy change (ΔGd) for the unfolding reaction was calculated using equation 4 and extrapolating 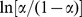 to zero denaturant concentration:
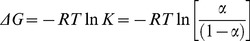
(4)where R is the gas constant (0.00198 kcal.g−1.mol−1) and T (298 K) is the absolute temperature in degrees Kelvin.

Samples (1 μM for the monomer and 0.5 μM for the dimer) were incubated with increasing concentrations of urea (0–6 M) and allowed to equilibrate for 2 h prior to performing measurements. Each experiment was performed at least three times with different protein preparations. To evaluate fold recovery upon chemical denaturation, a concentrated protein stock solution was initially prepared in 6 M urea and diluted to yield a 1 µM MpNep2 solution with urea concentrations ranging from 0.25–6 M. For the thermal experiments, MpNep2 (1 µM) was subjected to increasing temperatures in steps, with 10 min for temperature stabilization before Trp measurements. Fluorescence emission measurements were conducted as described above on an ISSK2 spectrofluorometer (ISS Inc., Champaign, IL) coupled with a Peltier temperature controller (Quantum Northwest – TC125). All samples and urea stock solution were prepared in 20 mM phosphate buffer (pH 7.4) containing 80 mM NaCl. Experiments were performed at 25°C.

### Circular dichroism (CD)

CD measurements were carried out on a Jasco spectropolarimeter (model J-715, 1505, Jasco, MD) equipped with a Peltier temperature controller and a thermostatted cell holder interfaced with a thermostatic bath. The far-UV spectra were recorded in a 0.2 cm path-length square cuvette at a protein concentration of 3 µM. CD spectra were measured at wavelengths ranging from 190 to 260 nm at 20°C resulting in an average of four spectra with a scanning speed of 100 nm.min^−1^ and a resolution of 0.1 nm for each step. The data were corrected for the baseline contribution of the buffer (20 mM phosphate buffer, pH 7.4, containing 80 mM NaCl). The thermal unfolding experiments were performed by increasing the temperature from 20 to 80°C at 1°C.min^−1^, allowing temperature equilibration for 5 min, and monitoring the content of the MpNep2 secondary structure at 222 nm during thermal denaturation.

### Dynamic light scattering (DLS)

DLS measurements were performed on a Brookhaven 90Plus/Bi-Mas Multiple angle particle sizing instrument (Holtsville, NY). Data were analyzed using the MAS Option software provided by Brookhaven. Each measurement consisted of at least 5 independent readings, and each reading was 30 seconds in duration. MpNep2 monomer (120 μM) and dimer (45 μM) in 20 mM phosphate buffer containing 80 mM NaCl (pH 7.4) were harvested at 10,000 x*g*, 10 min, 4°C and filtered (0.22 μm). All measurements were carried out at 25°C.

### Differential scanning calorimetry (DSC)

DSC measurements were performed using a VP-DSC calorimeter from MicroCal, LLC (Northampton, MA) and 10, 20 or 30 μM MpNep2 in 20 mM phosphate buffer (pH 7.4) containing 80 mM NaCl. Samples were degassed extensively before the experiment. DSC scans were performed between 20 and 60°C at a scan rate of 60°C.h^−1^, with a prescan thermostat of 10 min and a filter time of 16 s. The reference cell was filled with the same buffer as that used for the sample. Prior to data analysis, baselines were determined and substracted from the protein scans, which was followed by data normalization for protein concentration. To test thermal denaturation reversibility, MpNep2 solutions were cooled *in situ* to 20°C for 5 min immediately after the first scan was completed (ranging from 20 to 60°C) and rescanned one or more times under the same experimental conditions. Usually, two or four successive identical DSC scans were performed before the analysis of individual scans.

### Small-angle X-ray scattering (SAXS)

SAXS data were collected at the SAXS2 small-angle scattering beamline of the National Synchrotron Light Laboratory, Campinas, Brazil, using a two-dimensional MARCCD detector with a mica sample holder and a wavelength *λ* of 0.1488 nm at 25°C. Experiments were performed in 20 mM phosphate buffer (pH 7.4) containing 150 mM NaCl at 5 mg.mL^−1^ for the MpNep2 dimer and 2.5 mg.mL^−1^ for the MpNep2 monomer. Data acquisition was performed by taking three frames of 300 sec for each sample. The modulus of scattering vector *q* was calculated according to the relationship 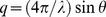, where *λ* is the wavelength used and 2*θ* is the scattering angle. The sample-detector distance was set to allow detection of a *q* range from 0.0176 to 0.45 Å^−1^. The monodispersity of the samples was confirmed by Guinier plots of the data. Data were corrected properly and fitted using GNOM [26], and the low-resolution *ab initio* particle shape was restored using GASBOR [27]. The final model was the result of an average of ten independent calculations performed using DAMAVER [28]. To create superposition models, we first used SUPCOMB software [29] to obtain the best fit orientation between the crystal structure of MpNep2 (PDB entry: 3ST1) and the shape reconstruction of the monomer followed by manual adjustment of this superimposed model with the shape reconstruction of the dimer using Pymol Executable Build software (based on Pymol v.099).

### Nuclear magnetic resonance (NMR)

A complete data set for the NMR experiments composed of TROSY-based 3D triple resonance HNCACB, HNCA, HNCO, CBCA(CO)NH and HN(CA)CO spectra were collected for sequential backbone resonance assignment of the monomer (100–250 μM) in 20 mM phosphate buffer (pH 7.4) containing 80 mM NaCl at 25°C using a Bruker Avance III at 800 MHz. The assignments were confirmed and completed with ^15^N-edited NOESY-HSQC and ^13^C-edited NOESY-HSQC spectra (both using 120 ms mixing time). All spectra were processed using Topspin 2.0. For chemical-shift perturbation (Δ*δ*) analysis, ^1^H-^15^N resonance variations were obtained from ^15^N-HSQC experiments with the MpNep2 monomer (100–250 μM) and dimer (100–250 μM) free in solution. Shifted resonances were confirmed by careful analysis of the superposition spectra of the monomer and dimer. Data processing was performed with the CARA 1.8.4 program [30] using equation 5:
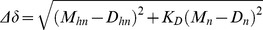
(5)where *M_hn_*, *D_hn_* and *M_n_*, *D_n_* are the ^1^H-chemical shift and ^15^N-chemical shift values for the MpNep2 monomer and dimer, respectively. A scaling factor (*K_D_*) of 0.1 was used.

## Results

### MpNep2 forms a dimer in solution

**Figure 1:**
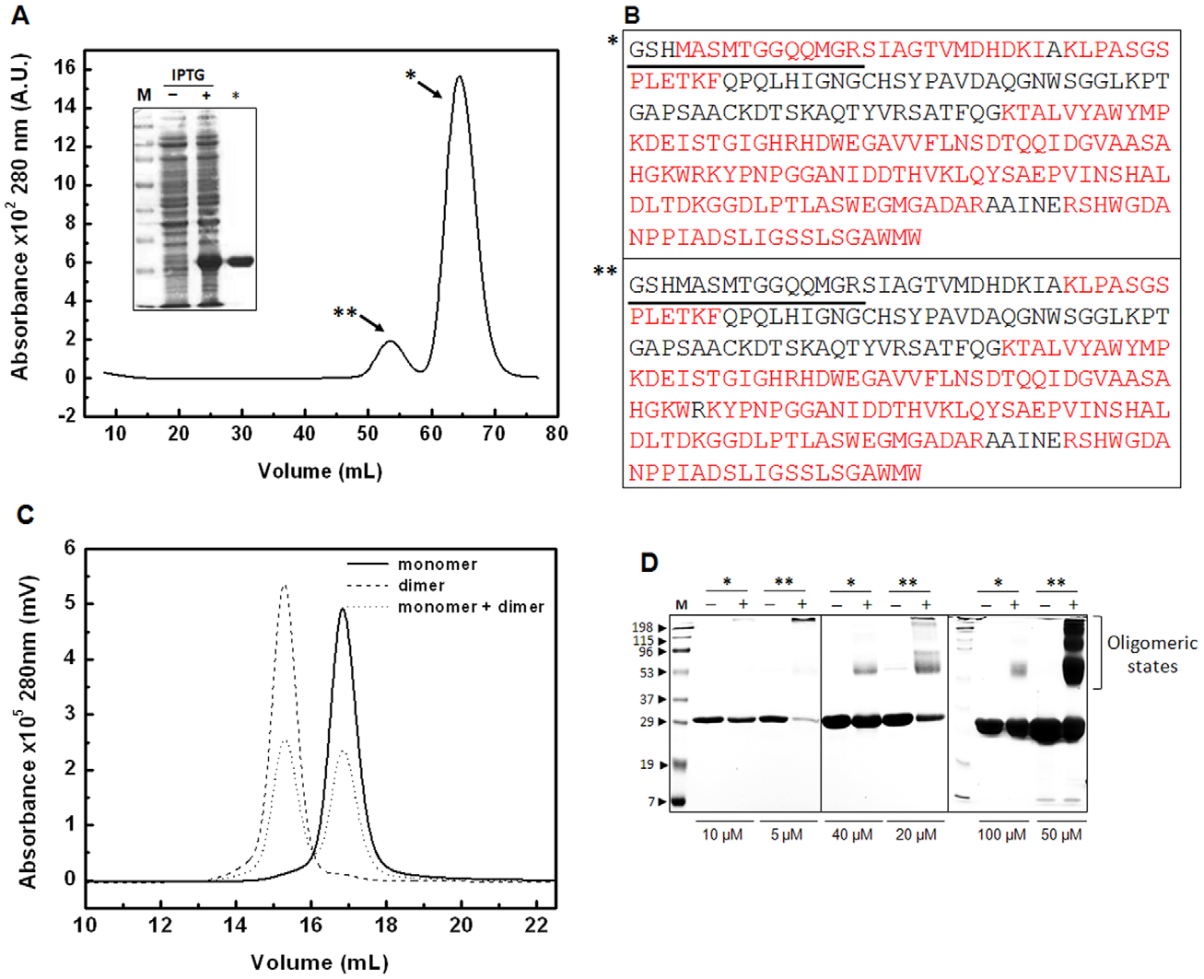
MpNep2 dimers co-exist with monomers. **A**. Preparative size-exclusion chromatography performed on Sephacryl S100 16/60 showed eluted peaks that correspond to MpNep2 monomer (*) and MpNep2 dimer (**). Inset shows a representative 15% SDS-PAGE of the purification protocol. (−) lane shows protein extract before IPTG induction and (+) lane show protein extract 16 h after induction with 1 mM IPTG. (*) lane shows MpNep2 monomer after purification. **B**. Protein coverage by electron-spray mass spectrometry is shown for MpNep2 monomer (*) and dimer (**). The entire MpNep2 sequence expressed by pET28a vector is shown. Common tryptic peptides are highlighted in red, cloning artifacts are underlined, and non-detected peptides are in black. **C**. Analytical size-exclusion chromatography of MpNep2 monomer (solid line), dimer (dashed line) and a mixture with equal amounts of each (dotted line) was performed on Superdex 200. Preparative and analytical chromatography at 0.7 mL.min^−1^ were monitored at 280 nm. **D**. 15% SDS-PAGE following glutaraldehyde cross-linking of MpNep2 monomer (*) and dimer (**) at different concentrations. (−) lanes show controls without glutaraldehyde and (+) lanes show cross-linked proteins after 5 min with 0.1% glutaraldehyde at 37°C. Cross-linking reactions were performed separately with equal amounts between monomers and dimers. 10, 40 and 100 μM were used for monomers and 5, 20 and 50 μM were used for dimers.

Highly homologous NLPs form oligomeric states that dictate functionality in the host cell [23]. During WBD progression, MpNep2 normally increases in concentration [23], a condition that should favor oligomer formation. Accordingly, we aimed to investigate MpNep2 oligomeric states in solution upon heterologous over-expression in an *Escherichia coli* strain. Recombinant polypeptide production in a heterologous system may lead to transient oligomerization patterns, so we take advantage of this fact to produce oligomeric species of MpNep2 for further characterization. Notably, the purification of recombinant MpNep2 revealed a minor peak eluted during the size-exclusion step performed on Sephacryl S100 16/60 (Fig. 1A). To rule out a possible artifact or contaminant protein, we found using electron-spray ionization mass spectrometry the same profile for tryptic peptides in the major and minor peaks (Fig. 1B and Table S1), suggesting that our purification protocol was able to isolate two MpNep2 conformers. UFLC of the purified conformers confirmed the retention volumes expected for proteins of ∼24 kDa and ∼48 kDa (Fig. 1C), and hydrodynamic radius (R_H_) measurements using DLS were in agreement with the expected molecular mass for the MpNep2 monomer and dimer (Table 1).

**Table 1:** Structural parameters for MpNep2 monomer and dimer.

MpNep2	SAXS Data			DLS Data
	1R_g__Guinier_ (Å)	R_g__Gnom_ (Å)	D_max_ (Å)	R_H_ (Å)
Monomer	21.02	19.7	65	25
Dimer	29.04	27.7	90	33

1 R_g_ was obtained from the angular coefficient (*a*) of the linear regression of the Guinier domain 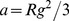.

Because chemical cross-linking can sometimes reveal protein-protein interactions *in vitro*, we conducted a cross-linking experiment with glutaraldehyde. Reactions with samples from both isolated peaks showed different susceptibilities to oligomer formation (Fig. 1D). During polypeptide enrichment, we were able to isolate the monomeric and dimeric fractions of MpNep2, but no oligomeric species could be observed other than those observed in the cross-linking experiment. These results show that MpNep2 may self-associate to form a homo-dimer *in vitro* upon over-expression in a heterologous system.

### MpNep2 dimer characterization

**Figure 2:**
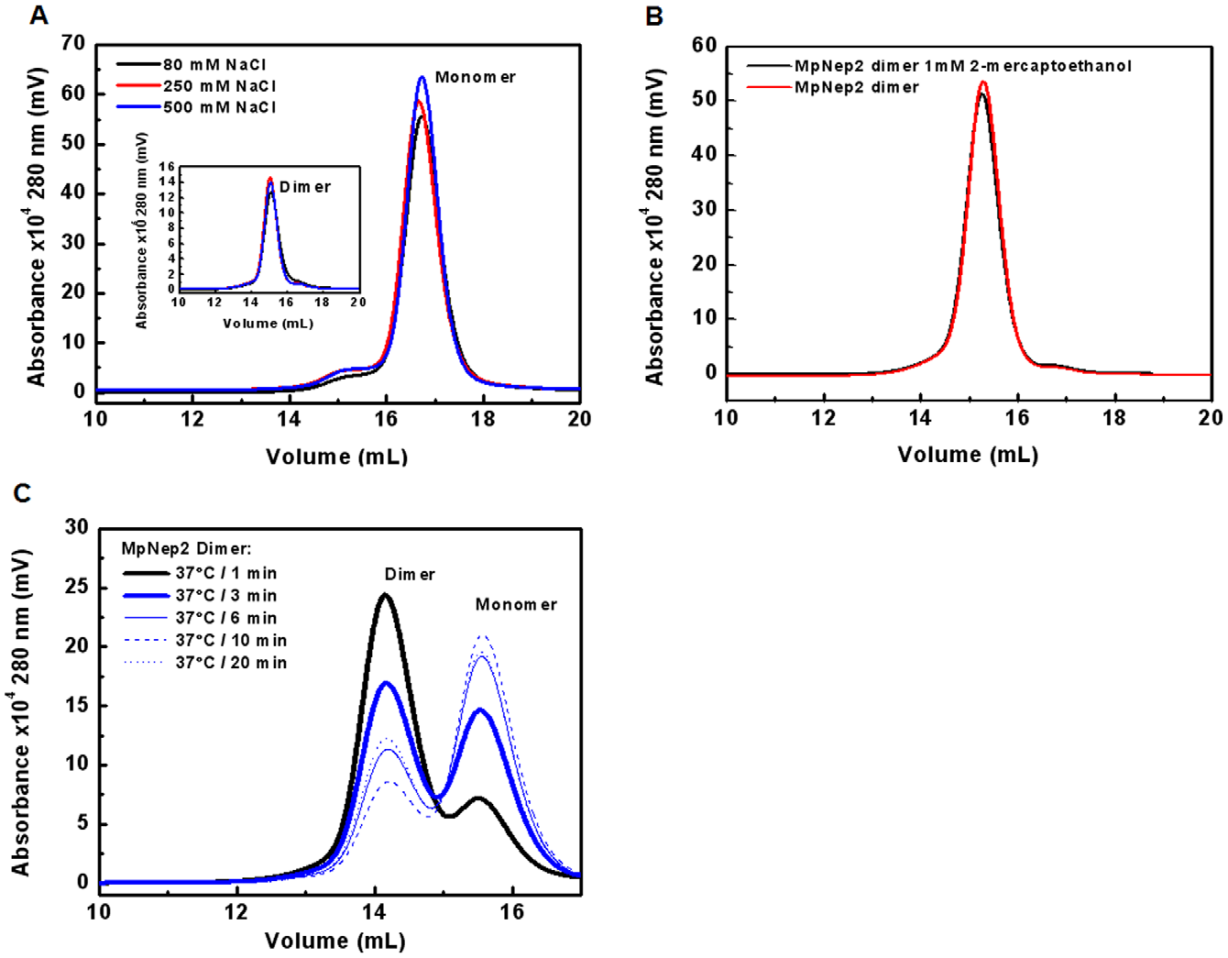
Analysis of MpNep2-MpNep2 interaction. **A**. UFLC performed at different ionic strengths for MpNep2 monomer (main painel) and dimer (inset). **B**. UFLC of MpNep2 dimer immediately performed after treatment with 1 mM 2-mercaptoethanol (black line). Non-treated protein is depicted as red line. **C**. UFLC of MpNep2 dimer after exposure to 37°C from 1–20 min. Traces were recorded at 280 nm and 0.7 mL.min^−1^ using Superdex 200.

Because dimerization was not previously reported for this NLP isoform, we performed experiments to further characterize the nature of the MpNep2-MpNep2 interaction. UFLC assays revealed that dimers were not sensitive to increases in ionic strength (Fig. 2A) or cysteine oxidation (Fig. 2B). Serial dilution of the MpNep2 dimer in the range of 30.4 μM to 3.4 pM was performed and monitored using a UFLC fluorescence detector. Dimer dissociation was not dependent on protein concentration in the range analyzed and during the time frame of the experiment (not shown). Although the MpNep2 dimer was stable at 25°C over 48 hours (not shown), a slight increase in temperature was able to dissociate it. To obtain further information about this transition, we performed a kinetic assay coupled with UFLC analysis. More than a half of the dimers were dissociated to monomers within 10 min of incubation at 37°C (Fig. 2C) and were not able to return to the dimeric state. These results suggest that MpNep2 dimerization is not *at equilibrium* and relies on a metastable state that can be disassembled by a slight input of energy to the system.

### SAXS and NMR reconstructions of MpNep2 monomers and dimers

**Figure 3:**
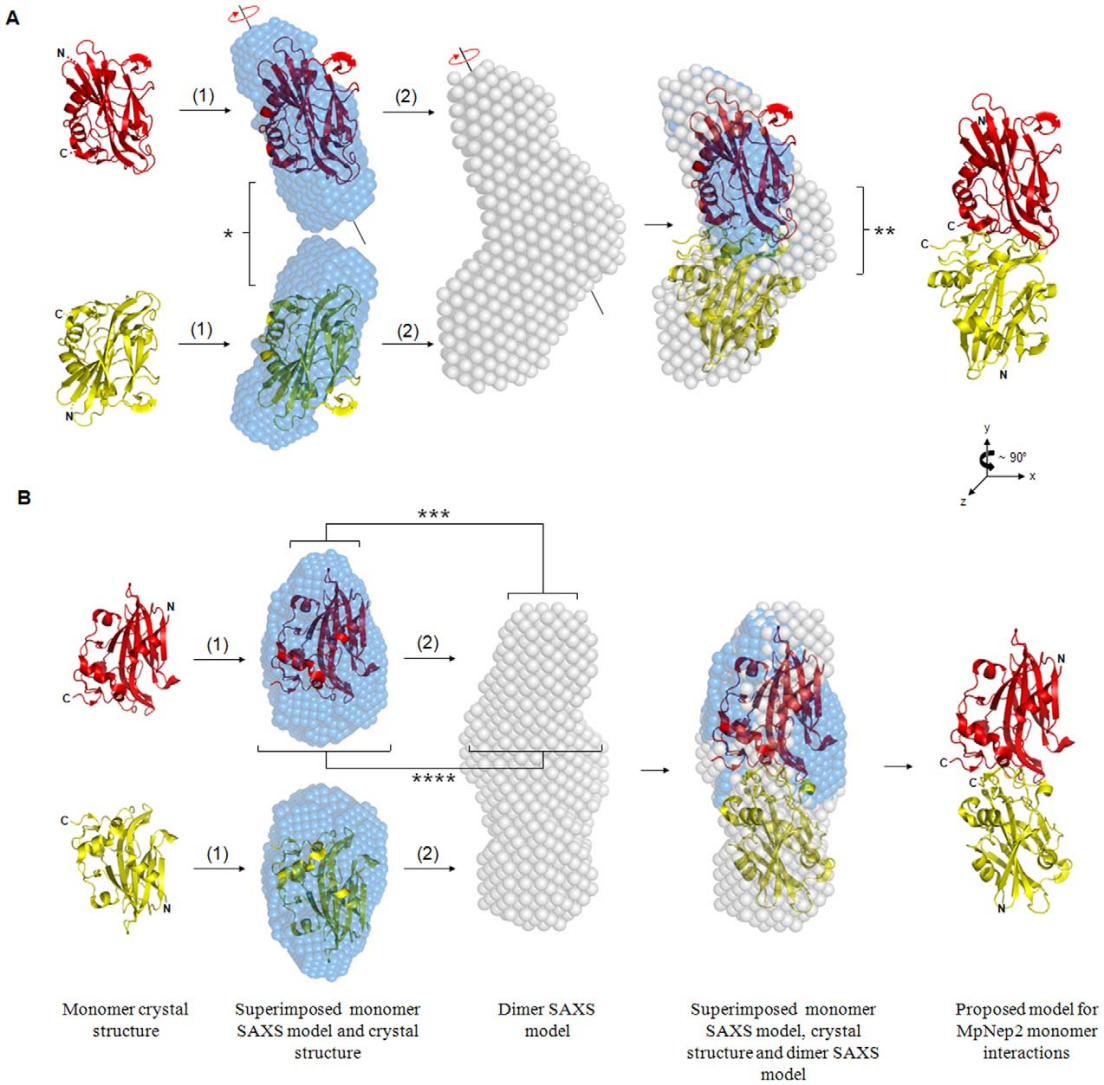
Proposed tail-to-tail mechanism of MpNep2 monomers interaction. **A.** Schematic representation of the monomer (blue) and dimer (light gray) molecular envelopes obtained by SAXS and the superposition procedure employed among the low and high resolution models. (1) Superposition between the crystal structure (PDB entry: 3ST1 – red and yellow) and the shape reconstruction of the monomer were first performed by SUPCOMB software and refers to the best fit obtained by the minimization software. (2) Superposed model obtained in (1) were manually adjusted in the shape reconstruction of the dimer to produce the best fit between the three models. * evidence an extra volume in the monomer SAXS model that was unacounted by the crystal structure. ** shows the packing conformation in the connecting interface of interaction between the monomers. The circular red arrows depicted in the top of the main axis of the models refer to the only alternative orientation we believe would be possible for the MpNep2 monomer during the dimeric state assembly. **B.** the same schematic representation as in A differing by 90° vertical rotation evidence the geometry of the monomer SAXS model that favored the manual adjustment in the dimer SAXs model using Pymol software. *** and **** show the narrower and broader density in the top and bottom of the monomer SAXS model respectively, that were particularly similar to the top and bottom density observed in the dimer SAXS model. All models are shown in the same orientation view in A and B differing between A and B, 90° vertical rotation.

To obtain further structural information on the dimer of MpNep2, we conducted experiments using SAXS. After obtaining the intensities of the scattering vector *q* [I(q)] (Fig. S1), the three-dimensional *ab initio* molecular envelope was constructed using a simulated annealing procedure with the program GASBOR [27]. Ten independent simulated annealing runs were conducted for each set of monomer and dimer X-ray data, and the resulting models were averaged with DAMAVER [28] to generate a consensus molecular envelope (Fig. 3A – blue for monomer and light gray for dimer). To assess the precision of the consensus structure, the normalized spatial discrepancy parameter was obtained, which provides a quantitative estimate of the similarity between different models obtained by simulated annealing. Low values of the normalized spatial discrepancy (∼1.0) indicate a good agreement between individual models [28]. The mean value of the normalized spatial discrepancy for ten separate runs was 0.835±0.015 for the monomer and 0.933±0.017 for the dimer. The consensus shape reconstruction for the MpNep2 monomer revealed an oblong shape (Figs. 3A and B), and the superimposed models of the monomer and the dimer (see material and methods) revealed a possible tail-to-tail interaction between the monomers (Figs. 3A and B). Several other orientation trials using the superimposed model of the crystal structure with the monomer molecular envelope and the dimer low-resolution model were performed by manual adjustment, with none of them leading to a coherent superposition. Table 1 summarizes the radius of gyration (R_g_) and maximum distance (D_max_) from end-to-end along the longest axes for each form. A comparison between the R_g_ obtained by SAXS and the R_H_ obtained by DLS revealed a higher R_H_ for the monomer and dimer, possibly due to hydration effects on the protein. The proportional increase in the values of R_H_ compared to those of R_g_ may also reflect that monomer assembly did not disturb protein hydration.

**Figure 4:**
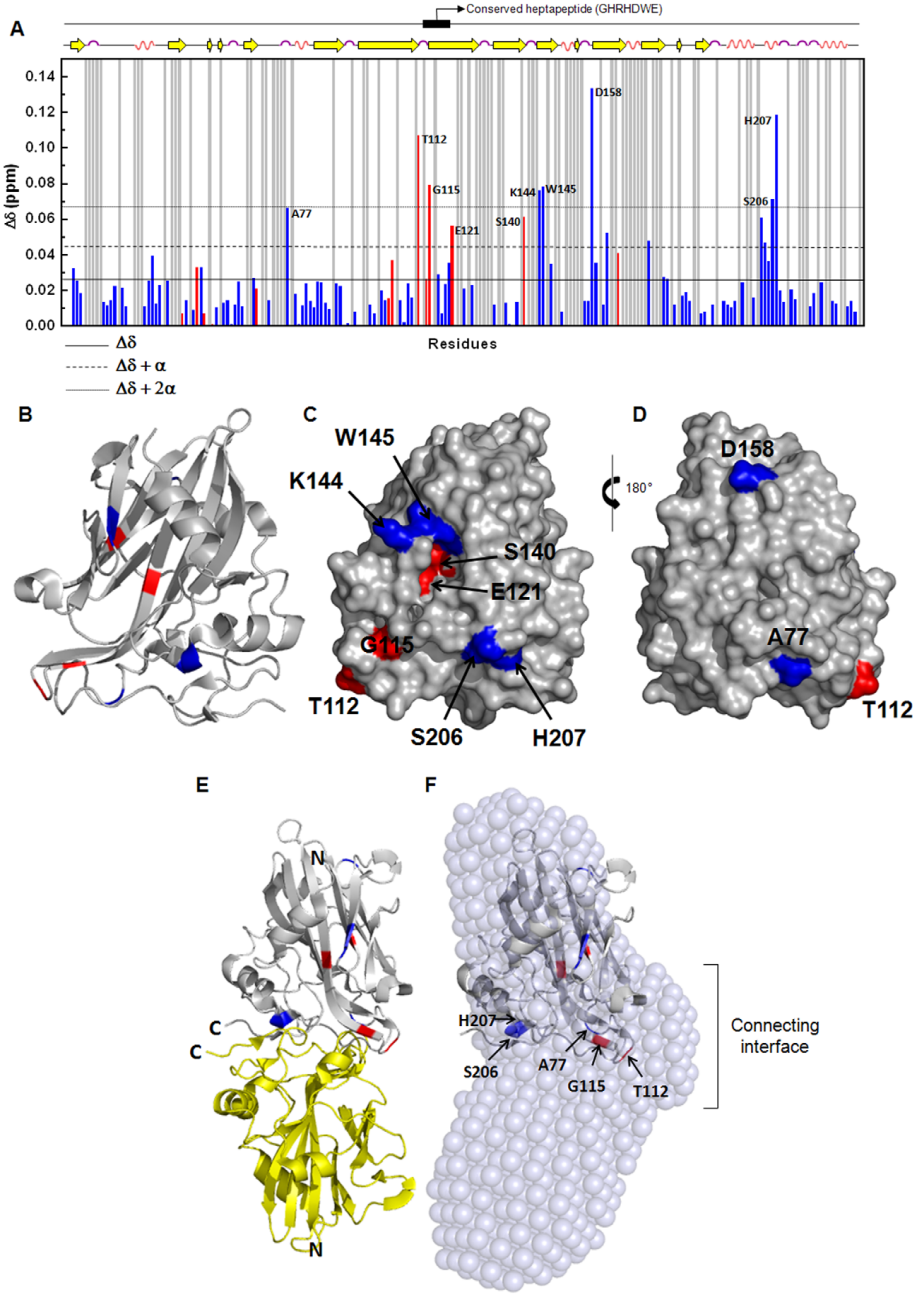
MpNep2 dimer interaction sites identified by NMR. **A**. Chemical-shift perturbation (Δ*δ*) plot as a function of residue shows the most exchangeable residues between MpNep2 monomer and dimer. The statistical significance of the change seen for an individual residue can be judged from the horizontal lines: solid, dashed and dotted lines comprise Δ*δ* mean from analyzed residues, Δ*δ* mean plus one time standard deviation (α) and two times standard deviation, respectively. Gray columns refer to residues that were not included in Δ*δ* analysis due to superposition or no assignment. Red and blue columns refer to residues that were shifted and decreased in intensity and only shifted from the HSQC spectra comparison, respectively. Secondary structure and heptapeptide position is depicted in the top of the plot. **B**. Ribbon representation of MpNep2 monomer. **C** and **D** Surface representation of MpNep2 monomer in two views differing by a 180° vertical rotation. Colors in B, C and D have the same significance as in A. **E**. Ribbon representation of MpNep2 dimer built after superposition with SAXS data. **F**. Superposition of MpNep2 monomer ribbon representation with the dimeric model obtained from SAXS reveals that five perturbed residues observed by NMR are located into the interface of interaction between the monomers.

Finally, after backbone resonance assignment of the MpNep2 monomer (∼94% of the whole sequence), we performed ^1^H-^15^N HSQC experiments on both MpNep2 conformers to obtain additional information on the possible tail-to-tail interface. Comparison of ^1^H-^15^N HSQC spectra revealed similar crosspeak dispersion for the MpNep2 monomer and dimer (Fig. S2). However, chemical-shift perturbation (Δ*δ*) analysis showed that 10 residues (Ala77, Thr112, Gly115, Glu121, Ser140, Lys144, Trp145, Asp158, Ser206 and His207) were significantly shifted between the monomer and dimer conformations (Fig. 4A). Residues were mapped onto the MpNep2 crystal structure (PDB entry: 3ST1), and five of them (Ala77, Thr112, Gly115, Ser206 and His207) were located in the putative interface between monomers derived from the SAXS data. The acidic residue Glu121 is located in a negatively charged cavity that has previously been described [20], and Ser140, Lys144 and Trp145 are located in the nearby vicinity of this cavity comprising the base of the turn between β-strand 7 and 8. Asp158 is located in the opposite side of these perturbed residues (Figs. 4B-F).

### Thermodynamic characterization of the MpNep2 monomer and dimer

**Figure 5:**
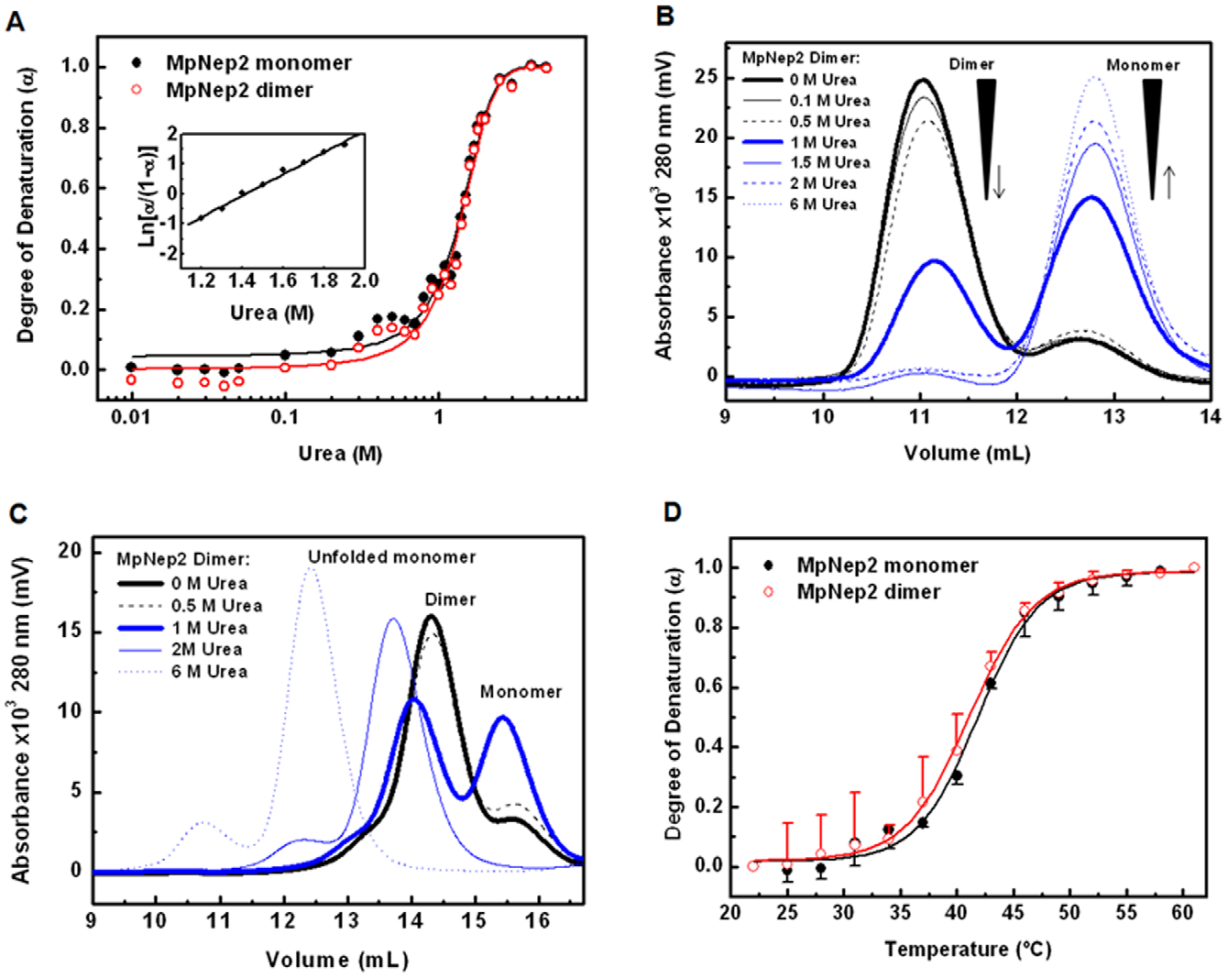
Thermodynamic characterization of monomeric and dimeric MpNep2 by chemical and physical agents. **A**. Urea denaturation plots obtained by monitoring Trp fluorescence emission and calculating as degree of denaturation (*α*) from equations 1 and 2 (see Material and methods). Inset shows linear regression of 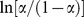
versus urea. **B**. MpNep2 dimer treated with different urea concentrations and recorded by UFLC with a running buffer that do not contain urea. **C**. MpNep2 dimer treated with different urea concentrations and recorded by UFLC with a running buffer containing the corresponding urea concentration depicted on legend. **D**. Thermal denaturation plots obtained by monitoring Trp fluorescence emission and calculating degree of denaturation. A and D – Experiments are shown as mean (n = 3) with independent protein preparations. B and C – UFLC was performed on Superdex 75 and Superdex 200, respectively at 0.7 mL.min^−1^.

Because MpNep2 has the ability to refold spontaneously *in vitro* after heating, we conducted physical and chemical thermodynamic experiments. Chemical denaturation curves, which monitor the changes in the spectral center of mass of the intrinsic Trp fluorescence emission from the monomer, revealed a cooperative fold for MpNep2, as observed by the *m*-value (Table 2). The magnitude of the *m*-value is indicative of the cooperativity of a two-state unfolding/refolding process. Significant changes in the center of spectral mass values were observed for concentrations of urea above 1 M, as evidenced by converting the data into the degree of denaturation (Fig. 5A). The results suggest a low stability for MpNep2 under this treatment. The superimposed urea denaturation plots of the monomer and dimer in figure 5A show that the MpNep2 dimer is quickly dissociated and unfolded following this treatment. Inspection of the individual curves yielded denaturant concentrations of 1.41 M at the midpoint of the transitions (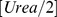) for the monomer and dimer. The treatment of the MpNep2 dimer with 1.5 M urea was sufficient to dissociate it almost completely to monomers based on the UFLC data (Fig. 5B). The refolding of MpNep2 to native monomers can be observed by comparing the UFLC plots with (Fig. 5C) and without (Fig. 5B) the addition of the corresponding urea concentration in the running buffer. This result clearly suggests that native and unfolded monomers exist *at equilibrium*, whereas the dimeric form of MpNep2 does not, as we were not able to detect a dimeric population using UFLC data after the withdrawal of urea from the running buffer. Thermal denaturation experiments also confirmed the dissociation/denaturation profile of dimer (Fig. 5D).

**Table 2:** Thermodynamic parameters for MpNep2 monomer and dimer.

MpNep2	Fluorescence Data				DSC Data	
	[Urea]/2 (M)	ΔG (kcal.mol^−1^)	*m* (kcal.mol^−1^.M^−1^)	Tm (°C)	ΔH (kcal.mol^−1^)	Tm (°C)
Monomer	1.41	2.85±0.12	2.10±0.07	41.8	109±7	42.2±0.2
Dimer	1.41	-	-	40.7±1.3	-	41.8±0.2

**Figure 6:**
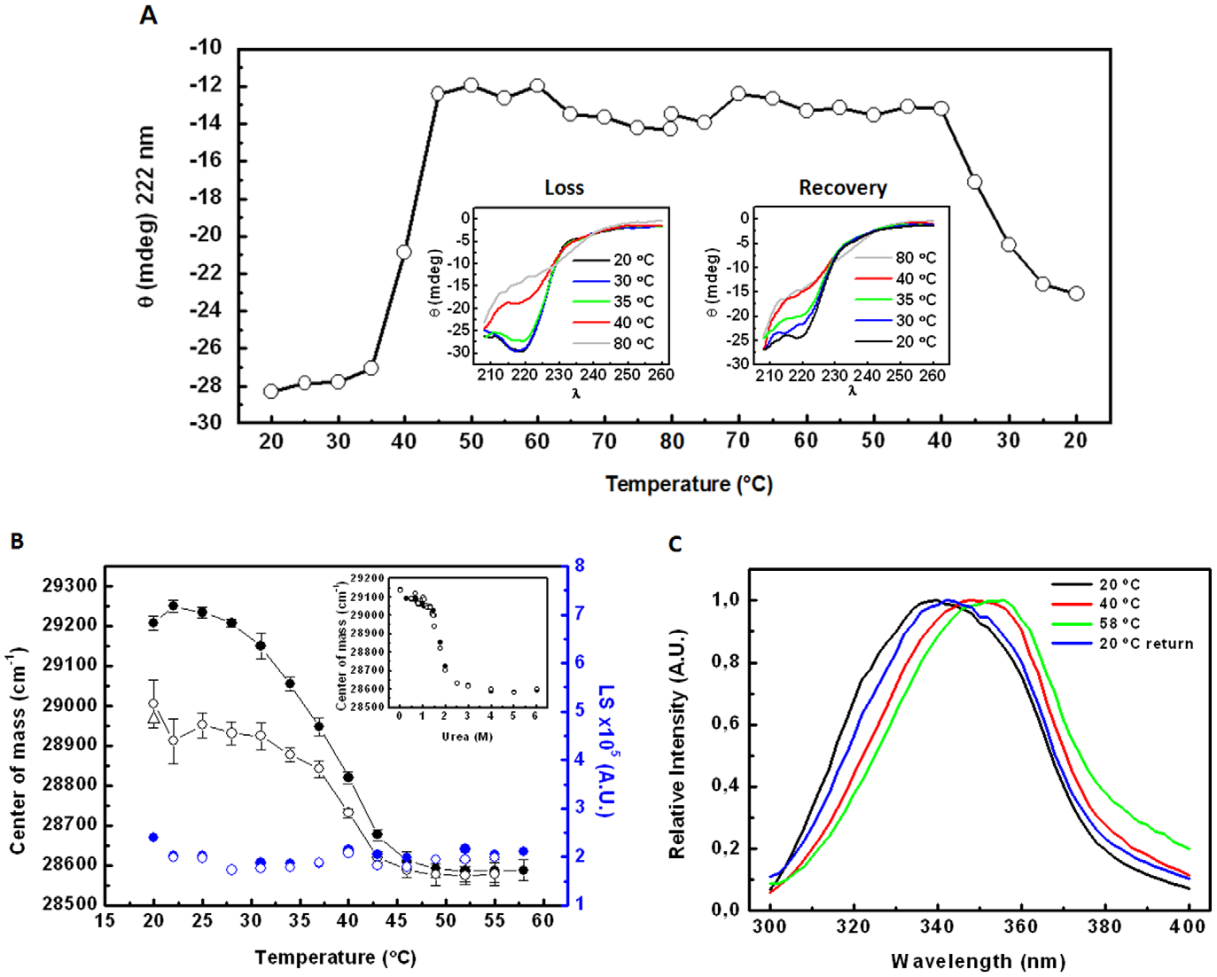
Denaturation/renaturation profile of MpNep2. **A**. Thermal denaturation plot of MpNep2 monomer monitoring 222 nm absorbance by circular dichroism. Right and left insets show far-UV scans from 260 to 210 nm obtained during the heating and cooling experiment, respectively. **B**. Thermal denaturation plot of MpNep2 monomer monitoring the center of spectral mass of the Trp fluorescence emission during the heating (black filled circles) and cooling (black open circles). Open triangle shows the center of mass after 1 h of cooling. Light scattering during heating (blue filled circle) and cooling (blue open circles) were also recorded. Inset shows urea reversibility plot monitoring center of mass of the Trp fluorescence emission. For thermal denaturation, proteins were left for 10 min in each temperature before recording the Trp spectra. **C**. Relative Trp fluorescence emission spectrum at 20°C, 40°C, 58°C and 1 h after cooling to 20°C. Experiments are shown as mean (n = 3) with independent protein preparatoins.

Thermal denaturation/renaturation plots based on circular dichroism and Trp fluorescence of the monomer revealed 81.5% secondary content recovery at 222 nm (Fig. 6A) and 99.3% recovery in the values of the center of spectral mass (Fig. 6B) from the Trp emission spectra (Fig. 6C); however, it was completely refolded when reversibility was tested by urea (Fig. 6B – inset). A comparison of far-UV spectra at 20°C revealed a similar shape in the helical region before and after the heating procedure (Fig. 6A – insets) but indicated a slight increase in ellipticity in the region lower than 215 nm for the recovered protein. Further investigation of this region was limited due to a signal-to-noise problem, but the increase in ellipticity at wavelengths lower than 215 nm is an indication that temperature may lead to discrete changes in the secondary content of the protein. Light scattering measurements rule out protein aggregation at 1 μM during thermal denaturation experiments for the monomer and dimer (Fig. 6B). These results suggest that, when exposed to heating, monomers assemble into a fold that incorporates discrete changes in the secondary and tertiary structure. Dimerization does not lead to changes in the secondary structure as evidenced by circular dichroism spectra (not shown).

**Figure 7:**
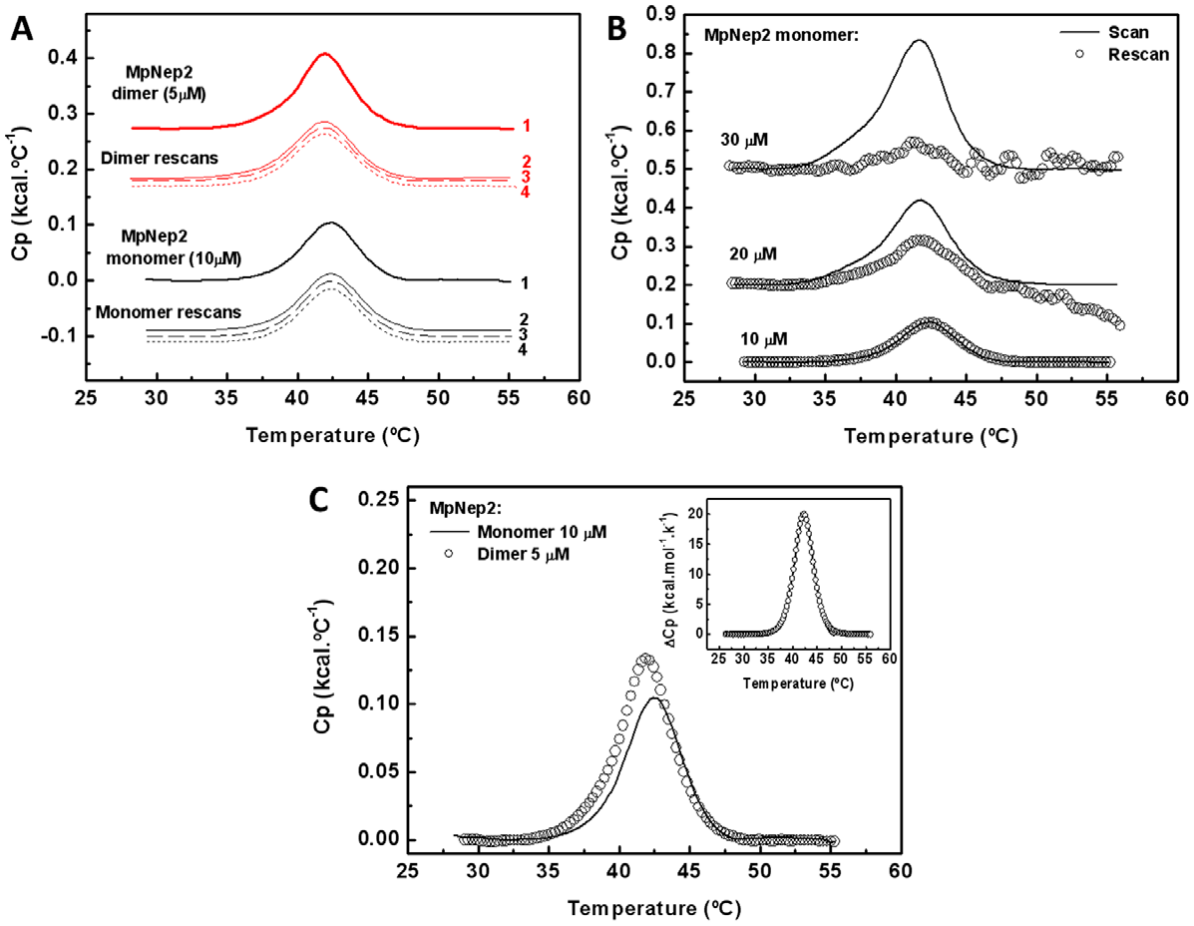
Comparative studies of the thermal stability of MpNep2 monomer and dimer by differential scanning calorimetry. **A**. Thermal unfolding reversibility of monomer and dimer was monitored by successive identical DSC scans. Depicted numbers (1 to 4) indicate the order of successive scans for monomer and dimer. **B**. Dependence concentration analysis of MpNep2 unfolding reversibility after identical DSC rescans (20 to 60°C) in this condition. Concentrations are those depicted in the plots. Solid lines and open circles show the first and second scans for each analyzed concentrations, respectively. **C**. Thermal unfolding plots for MpNep2 monomer and dimer are shown as solid line and open circles, respectively. Inset shows experimental thermogram of monomer normalized by concentration. Solid line and open circles represent experimental DSC scan and the fitting-by-model “independent two-state transition”. All thermograms are shown after baseline substraction.

To investigate the thermal stability of the MpNep2 monomer and dimer, we used DSC. Our results showed a single transition during thermal denaturation for the monomer. The area under the DSC scan provides the calorimetry enthalpy of unfolding, which decreases during successive scans of the sample in the case of irreversibility. At 10 μM of monomer, the thermal unfolding was completely reversible, as verified by the maintenance of calorimetry enthalpy after consecutive identical scans (20 to 60°C) (Fig. 7A and 7B). However, for the dimer at 5 μM under the same conditions, the area under the peak after consecutive scans was reduced by approximately 20% compared to the first scan. Thus, the transition for the dimer appears to be only partially reversible, most likely due to refolding into the monomeric form. This interpretation is supported by the kinetic assay coupled with UFLC analysis (Fig. 2C). We were not able to observe any evidence of protein aggregation after the fourth scan at 10 μM of the monomer or 5 μM of the dimer. On the other hand, at higher concentrations (20 and 30 μM), the transition was only partially reversible, as evidenced by exothermic events that are usually correlated with the aggregation and precipitation of largely denatured proteins (Fig. 7B). In addition, MpNep2 solution at 20 or 30 μM became cloudy after heating. The thermal unfolding of MpNep2 under the examined conditions is a reversible process at lower concentrations; however, at higher concentrations (20 and 30 μM), MpNep2 revealed a tendency to form aggregates, most likely due to high temperatures after the transition.

**Figure 8:**
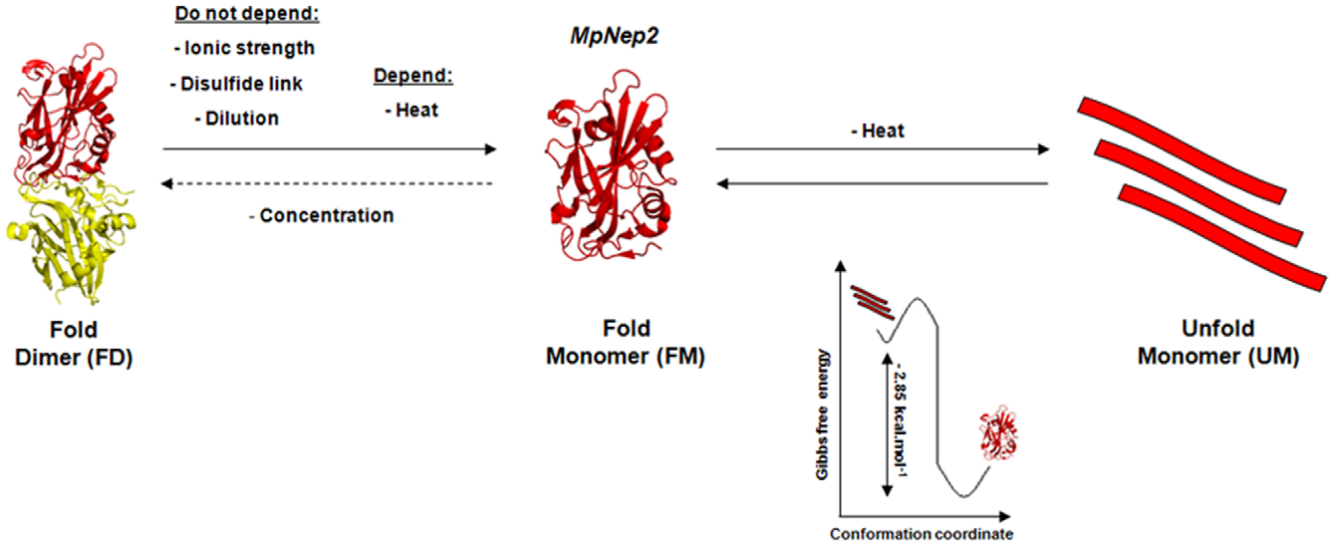
Schematic diagram showing observed features for MpNep2 transitions. Gibbs free energy diagram shows the conversion between unfolded and native MpNep2 monomer.

A comparative analysis of monomer and dimer thermal stability revealed additional heat associated with the dimerization process in this condition (Fig. 7C). For these measurements we used equivalent amounts of the monomer and dimer, 10 μM and 5 μM, respectively. The thermogram for the MpNep2 monomer showed a single peak with a melting temperature (T_m_) equal to 42.2°C and an unfolding enthalpy (ΔH) change of 109 kcal. mol^−1^ (Table 2). We were not able to obtain the unfolding enthalpy of the dimer due to the process being partially irreversible in this condition. The heat was not proportional to the amount of protein, as evidenced by additional endothermic events. Assuming a single transition to dimer unfolding, we found a T_m_ equal to 41.8°C. The interaction between monomers promotes a small reduction in T_m_ and an increase in calorimetric enthalpy (Fig. 7C and table 2).

Our results suggest that the MpNep2 monomer has a low stability and a cooperative mechanism for folding and that the refolded protein may recover its necrotic activity after heating with discrete changes in its secondary and tertiary structure.

## Discussion

Previous reports raised the issue that NLPs may form oligomers in solution, especially dimers [23], [31]. Additionally, it is well established that upon WBD progression, MpNep2 rather than MpNep1 accumulates in infected tissues, reaching its maximum concentration at the advanced necrotic stage [20]. Here, we have combined biochemical, thermodynamic and structural data to characterize the dimeric state of MpNep2. We also investigated the ability of the monomeric form to refold following heating.

For MpNep2 dimer production, we over-expressed MpNep2 polypeptide in a heterologous system. During the steps of recombinant polypeptide enrichment, transient oligomerization is common and may not have biological relevance. However, motivated by the accumulation of MpNep2 in advanced necrotic tissues and the propensity of NLPs to form dimers, we decided to take it into account to further characterize the dimeric form of MpNep2. In our purification protocol, we observed that dimer production occurred during His. Trap affinity chromatography and was enhanced by the concentration steps. Thus, MpNep2 dimer formation occurs in a concentration-dependent manner.

Using the scattering pattern of the MpNep2 monomer and dimer, we obtained the molecular envelope reconstructions for both conformers. The values of R_g_ were determined from the linear regression of the low *q* region of the Guinier plots and from the pair-distance distribution function of the interatomic vectors [P(r)] obtained with the software GNOM [26]. The measured values of R_g_ and D_max_ for the monomer (19.7 Å and 65 Å, respectively, from interatomic vector analysis) and dimer (27.7 Å and 90 Å, respectively, from interatomic vector analysis) agree well with the expected values for these polypeptides and are consistent with each other. The R_g_ and D_max_ values obtained from the dimer were not twice the values obtained for the monomer protein, confirming that dimeric MpNep2 exists in a compact conformation. This compact conformation can be observed by our superimposed models obtained from SAXS (see Fig. 3A). It is clear that the connecting interface between the monomers, mostly formed by loop regions, assembles together. This packing was also observed for the assembly of hetero-dimeric species [32].

The symmetry of the P(r) function provides insights to the particle's shape. The shape of the curve of the MpNep2 monomer is more symmetrically distributed, whereas the MpNep2 dimer is relatively skewed (Fig. S1), suggesting a more elongated particle in the dimeric state. The oblong shape reconstruction of the monomer matches the MpNep2 crystal structure very well (Fig. 3A and B). *Post-hoc* analysis of the monomer shape reconstruction shows additional density in the top and bottom regions that is unaccounted for in the crystal structure. As observed in the superimposed models, these extra volumes are presumably due to loop regions localized at the top and bottom of the crystal structure (see Fig. 3A). Additional volume was also observed in the dimer shape reconstruction in the connecting interface, which appears to be formed by the loop at the base of the β-sheet core of the protein. The superimposed models of the MpNep2 monomer, dimer and the crystal structure revealed a possible tail-to-tail interaction in the connecting interface. Based on our reconstruction data and superposition analysis, we assumed that no alternative orientation for the monomers would be favored in the dimeric conformation except for the rotation of the monomer around its main axis during the assembling of the dimeric state (see Fig. 3A, circular arrow). However, we did not address this hypothesis with experimental data. Additionally, because our SAXS data revealed that monomer interactions occurred via the C-terminus region, we infer that no residues in the N-terminus region, including the cloning artifact residues, would influence the dimerization process.

A chemical shift perturbation analysis of the ^1^H-^15^N HSQC spectra showed that 10 residues were significantly involved in the contact between monomers. Surprisingly, we observed that the conserved heptapeptide (GHRHDWE) was included in the putative connecting interface of the dimer and that Gly115 and Glu121 of the heptapeptide were significantly perturbed in chemical shift values and in the intensity of the peaks. Both peaks corresponding to Gly115 and Glu121 decreased in intensity in the dimer spectra. Thus, it is possible that the conserved heptapeptide motif participates in the dimerization process. The heptapeptide is a conserved sequence throughout evolution, so this result may demonstrate a mechanism of dimerization that is shared among members of the NLP family. It was previously reported that this motif comprises the bottom of a negatively charged cavity in the monomer [20], and mutational analysis reveals that it plays a role in MpNep2 necrotic activity [20]. Metastability has been involved in several protein misfolding diseases [33]. One possible hypothesis to explain our chemical-shift perturbation data is that the dimer of MpNep2 represents a reservoir of quiescent inactive protein in the apoplast of the infected host, in which the heptapeptide is buried by the MpNep2-MpNep2 interface of interaction. An increase in temperature or another environmental stress might lead to dimer dissociation, exposure of the heptapeptide, and consequently activation of MpNep2′s function. Necrotic activity would ensue following the triggering of cellular defense responses. Preliminary data have indicated that MpNeps are effectively present in the apoplast of infected tissues and appear to accumulate externally in the cacao plant cell wall. Additionally, it is remarkable that MpNep2 is present in biotrophic mycelia, which are present in cacao tissue for weeks without causing any apparent necrosis [2]. These data provide compelling evidence for the existence of an inactive form of MpNep2 in the apoplast of the infected host.

The oligomeric profile of MpNep2 has not been studied previously. Cechin and colleagues [31] discussed how NLPs could form oligomers based on sequence and structural similarities to the Cupin superfamily, a diverse group of proteins containing a conserved barrel domain known as *cupa*. MpNep2 protein was believed to act primarily in biotrophic mycelia as a monomer. However, MpNep1, a highly homologous NLP isoform, was shown to act ubiquitously as an oligomer in both biotrophic and saprophytic mycelia [23]. Because of these different expression profiles, MpNep1 and MpNep2 were thought to have complementary roles during disease progression [23]. However, this idea is not consistent with RNA-seq results in which only MpNep2 was expressed during the fungal infection [20]. One issue that remains unclear regards the role, if any, of MpNep1 and the other NLPs in WBD development. One hypothesis would be that these proteins undergo rapid evolution, as only a few essential conserved positions are required for the maintenance of architecture and activity [12]. Thus, the rest of the sequence would be prone to mutations and consequently the loss of protein function [12] and the formation of pseudogenes [9], [34].

The expansion of these NLP sequences (five identified NLPs) in the genome of *M. perniciosa* suggests the importance of this protein to the species and a possible mechanism for the transfer of function throughout evolution. MpNep1 and MpNep2 may be an example of this evolutionary mechanism, in which MpNep1 lost a function that was acquired by MpNep2 with additional properties, such as the ability to refold when exposed to temperature oscillations and the ability to form a metastable dimer. It is noteworthy that several microorganisms have more than one copy of NLP: the *Phytophthora spp*. genome has more than 40 copies of Nep [34], and in *P. megakatya*, a cacao pathogen that causes black pod disease in Africa, nine orthologs have been found, and at least six of them appear to be expressed [35].

Changes in the free energy of denaturation (Δ*G_d_*  = 2.85±0.12 kcal.mol^−1^) from the folded monomer (FM) to the unfolded monomer (UM) transition reveals that the MpNep2 fold is easily assembled and disassembled, suggesting a high tertiary structure flexibility. The *m*-value equal to 2.10±0.07 for the MpNep2 monomer is relatively high compared to other proteins [36]. Because monomeric MpNep2 spontaneously refolds and recovers its necrotic activity after thermal stress, a cooperative fold and high tertiary structure flexibility is a reasonable explanation for this observation. In figure 8, we summarize the main results obtained for MpNep2 transition states. Although the denaturation plots for MpNep2 dimers and monomers were quite similar, the MpNep2 dimer follows a non-two-state transition model in which the folded dimer (FD), folded monomer (FM) and unfolded monomer (UM) can be significantly populated. Native monomers were not populated in dimer denaturation, as the energy barrier between folded dimers and folded monomers may be extremely small. Because dimerization was not observed to be *at equilibrium*, we were not able to calculate the free energy change for dissociation/denaturation. However, DSC data showed that dimer unfolding required additional heat compared to monomer unfolding. The additional heat is most likely associated with the dimer dissociation in this condition. Moreover, the additional endothermic event appears to be related to the acceleration of the dimer dissociation process caused by a temperature increase, as also verified by UFLC analysis.

It is well known that during WBD progression, MpNep2 increases in concentration, but based on our knowledge, the quantification of a threshold concentration of secreted MpNep2 at the advanced necrotic stage, in which the dry broom symptoms are noticeable and MpNep2 expression reaches its maximum [20], still represents an Achilles heel. Thus, it is difficult to address any correlation between the MpNep2 concentrations reached in this study following over-expression in a heterologous system and those obtained *in planta* during an infection. Indeed, further research should be conducted to detect and prove any involvement of an oligomeric state in WBD progression.

In conclusion, we have provided evidence to explain the recovery of MpNep2 activity after heating and offered a model for the MpNep2 dimer structure. The formation of oligomeric species in NLP members, except in the case of MpNep1 [23], was not previously expected to be biologically relevant in phytopathological diseases, even with the knowledge that this group of proteins increases in concentration during disease progression, a hallmark for aggregation in biological systems. One possibility is that the dimer of MpNep2 is an inactive zymogenic state that can only be activated by thermal stress. We are currently attempting to test this possibility in a phytopathological model. We hypothesize that the proposed model described here should be taken into account to consider the involvement of these species in phytopathological disorders. The detection of NLP oligomers *in planta* and their involvement in the infection progress and spreading may open new avenues for the understanding of phytopathological diseases.

## Supporting Information

Figure S1 **SAXS scattering data for MpNep2 monomer and dimer.** **A**. Raw data for the scattered intensities versus *q* [I(q)] for MpNep2 dimer (cyan), monomer (light yellow) and the contribution from the buffer (light gray). **B**. Guinier plots. The linear regions of the low *q* section are shown as red lines (inset). **C**. Relative plot of the interatomic distances (P[r] function). In B and C values refer to the difference between protein solution and buffer contribution.
(TIF)

Figure S2 **^1^H-^15^N HSQC spectrum.** MpNep2 monomer (blue crosspeaks) and dimer (red crosspeaks) used for chemical shift perturbation analysis. Residues with significant shifts are highlighted.
(TIF)

Table S1 **Peptide match for MpNep2 monomer and dimer obtained by mass spectrometry (MS/MS).**
(DOC)
