# Supplementary figures and images for: Moniliophthora perniciosa Necrosis- and Ethylene-Inducing Protein 2 (MpNep2) as a Metastable Dimer in Solution: Structural and Functional Implications
Source: PLoS One. 2012 Sep 24;7(9):e45620. doi: 10.1371/journal.pone.0045620 (PMC3454426; doi:10.1371/journal.pone.0045620)

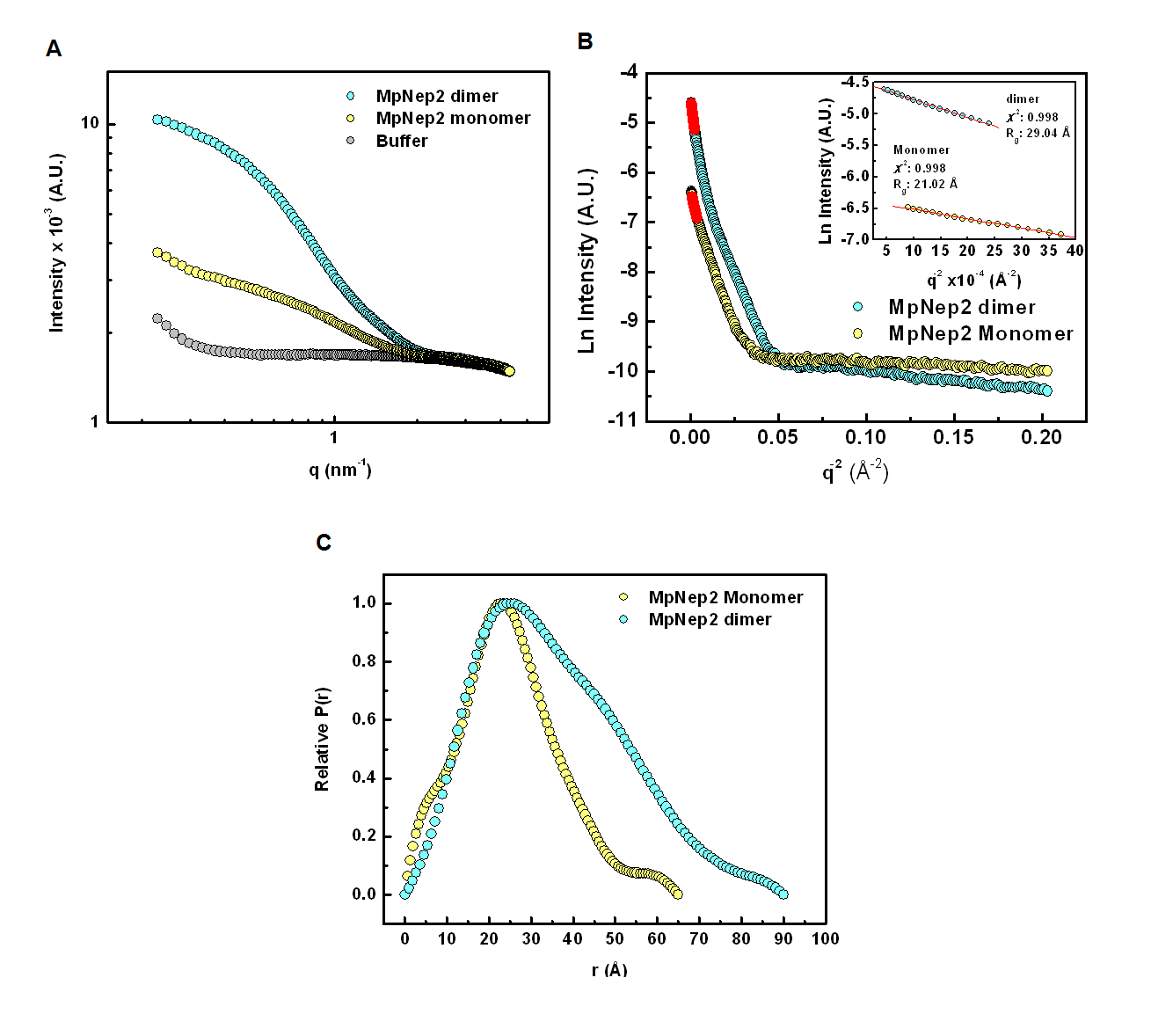

Supplement: Figure S1 — SAXS scattering data for MpNep2 monomer and dimer. A. Raw data for the scattered intensities versus q [I(q)] for MpNep2 dimer (cyan), monomer (light yellow) and the contribution from the buffer (light gray). B. Guinier plots. The linear regions of the low q section are shown as red lines (inset). C. Relative plot of the interatomic distances (P[r] function). In B and C values refer to the difference between protein solution and buffer contribution. (TIF) [file pone.0045620.s001.tif]

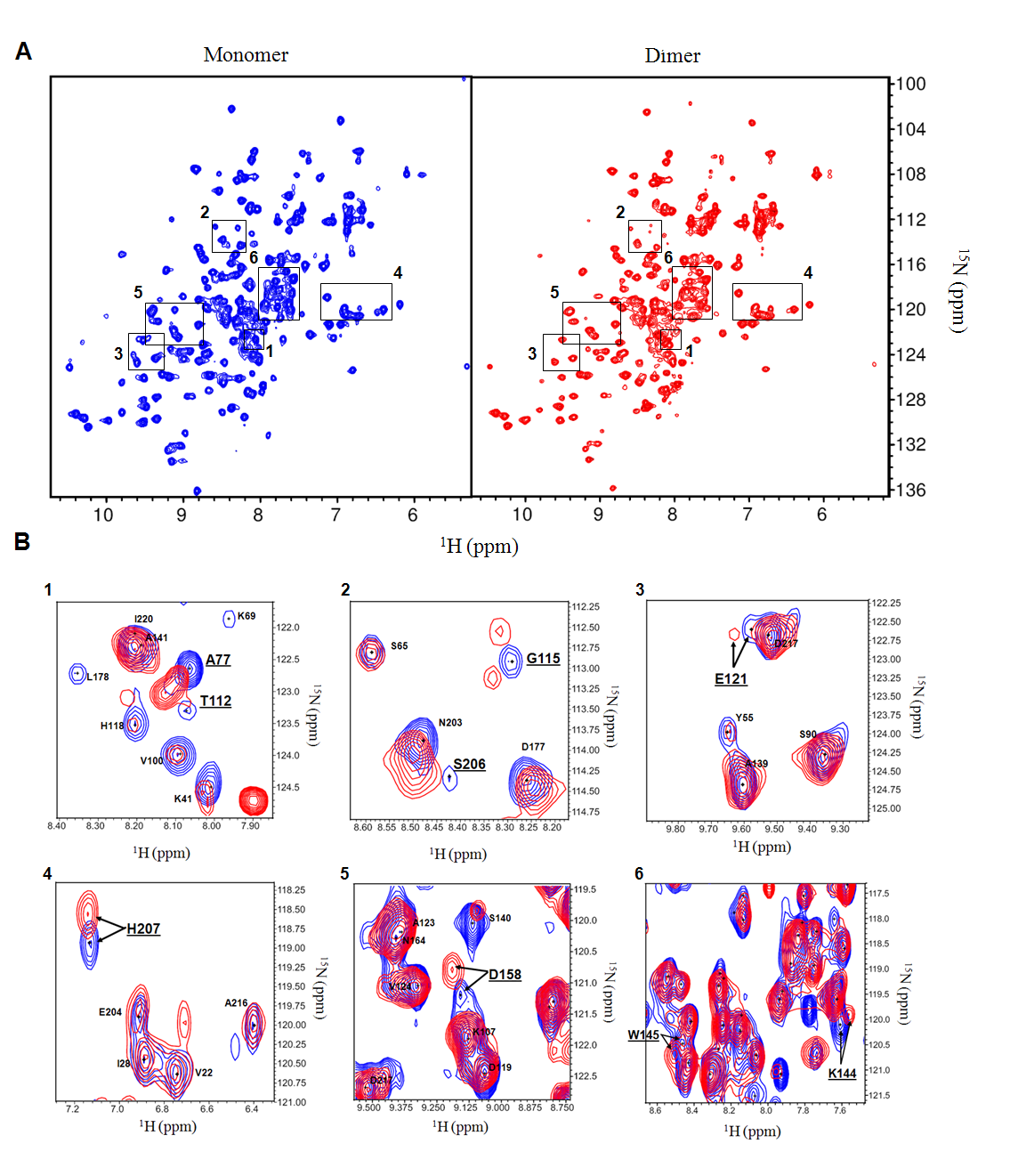

Supplement: Figure S2 — 1H-15N HSQC spectrum. MpNep2 monomer (blue crosspeaks) and dimer (red crosspeaks) used for chemical shift perturbation analysis. Residues with significant shifts are highlighted. (TIF) [file pone.0045620.s002.tif]
