# Supplementary material for: Moniliophthora perniciosa Necrosis- and Ethylene-Inducing Protein 2 (MpNep2) as a Metastable Dimer in Solution: Structural and Functional Implications
Source: PLoS One. 2012 Sep 24;7(9):e45620. doi: 10.1371/journal.pone.0045620 (PMC3454426; doi:10.1371/journal.pone.0045620)
Supplement: Table S1 — Peptide match for MpNep2 monomer and dimer obtained by mass spectrometry (MS/MS). (DOC) [file pone.0045620.s003.doc]

**Table S1**. Peptides matches for MpNep2 monomer and dimer obtained by Mass spectrometry (MS/MS).

| Peptide sequence | | Hits^#^ | Matched *m/z* (Difference) from search mass |
| --- | --- | --- | --- |
| Monomer * | MASMTGGQQMGR  KDEISTGIGHRH | 5  3 | 562.2496 (-0.0064)  542.7714 (-0.0026) |
|  | KLPASGSPLETKF | 4 | 550.2930 (-0.0206) |
|  | SIAGTVMDHDKI | 1 | 587.2792 (-0.0057) |
|  | KTALVYAWYMPKD | 3 | 671.8266 (-0.0405) |
|  | KYPNPGGANIDDTHVKL | 4 | 533.2524 (-0.0179) |
|  | RKYPNPGGANIDDTHVKL | 4 | 575.9392 (-0.0524) |
|  | KGGDLPTLASWEGMGADARA | 3 | 910.4130 (-0.0092) |
|  | KLQYSAEPVINSHALDLTDKG | 6 | 1057.5290 (-0.0257) |
|  | RSHWGDANPPIADSLIGSSLSGAWMW | 5 | 136.1128 (-0.0062) |
|  | RHDWEGAVVFLNSDTQQIDGVAASAHGKW | 11 | 713.8446 (-0.0033) |
|  | KLQYSAEPVINSHALDLTDKGGDLPTLASWEGMGADARA | 6 | 975.4531 (-0.1010) |
| Dimer ** | KDEISTGIGHRH | 1 | 542.7673 (-0.0108) |
|  | KLPASGSPLETKF | 6 | 550.3015 (-0.0036) |
|  | KTALVYAWYMPKD | 4 | 671.8489 (0.0041) |
|  | KYPNPGGANIDDTHVKL | 3 | 799.3590 (-0.0498) |
|  | RKYPNPGGANIDDTHVKL | 5 | 575.9539 (-0.0083) |
|  | KGGDLPTLASWEGMGADARA | 3 | 902.3638 (-0.1127) |
|  | KLQYSAEPVINSHALDLTDKG | 6 | 1057.5425 (0.0013) |
|  | KTALVYAWYMPKDEISTGIGHRH | 3 | 803.4077 (0.0018) |
|  | RSHWGDANPPIADSLIGSSLSGAWMW- | 18 | 885.7530 (0.0148) |
|  | RHDWEGAVVFLNSDTQQIDGVAASAHGKW | 36 | 713.8451 (-0.0013) |
|  | KLQYSAEPVINSHALDLTDKGGDLPTLASWEGMGADARA | 9 | 975.4896 (0.0450) |

^#^ Hits on Mascot sever - Peptide masses were entered into the local MASCOT server v4.1 for identification (http://www.matrixscience.com/).
